# Prevalence and characteristics of adults with fetal alcohol spectrum disorder in corrections: a Canadian case ascertainment study

**DOI:** 10.1186/s12889-018-6292-x

**Published:** 2019-01-09

**Authors:** K. McLachlan, A. McNeil, J. Pei, U. Brain, G. Andrew, T. F. Oberlander

**Affiliations:** 1 Department of Psychology, University of Guelph, 50 Stone Road East, Guelph, Ontario N1G 2W1 Canada; 2 Educational Psychology, University of Alberta, Edmonton, Alberta Canada; 3 Department of Pediatrics, University of British Columbia, Vancouver, British Columbia Canada; 4 Department of Pediatrics, University of Alberta, Edmonton, Alberta Canada; 5 Division of Developmental Pediatrics, Department of Pediatrics, University of British Columbia, Vancouver, British Columbia Canada

**Keywords:** Fetal alcohol spectrum disorder, Prevalence, Criminal justice, Correctional, Prenatal alcohol exposure

## Abstract

**Background:**

Individuals with fetal alcohol spectrum disorder (FASD) experience a range of cognitive, affective, and physical deficits following prenatal alcohol exposure. They are thought to be overrepresented in criminal justice settings. However, limited evidence is available to inform prevalence. We sought to estimate the prevalence of FASD in a Northern Canadian correctional population.

**Methods:**

Using an active case ascertainment approach we recruited a representative sample of 80 justice-involved adults (ages 18–40, 85% male) over an 18-month period from 2013 to 2015. Participants completed interdisciplinary clinical assessments comprising medical and psychological evaluations that adhered to the 2005 Canadian FASD Diagnostic Guidelines.

**Results:**

We identified a high rate of FASD (17.5, 95% CI [9.2, 25.8%]) in this sample, and this rate could have been as high as 31.2% with confirmation of prenatal alcohol exposure. Most participants in this study presented with significant neurodevelopmental and cognitive deficits in at least two domains of functioning, irrespective of diagnosis, with only five of 80 participants (6.3%) demonstrating no cognitive impairment.

**Conclusions:**

Findings showed disproportionately high estimated FASD prevalence in this representative sample compared to general population estimates in both Canada and the U.S. (2–5%), underscoring the need for improved FASD screening and diagnosis in correctional settings, and education for clinicians working in the justice context. Strengthened health prevention and intervention efforts to support the needs of individuals with FASD outside the criminal justice context are needed.

## Background

Fetal alcohol spectrum disorder (FASD) comprises the range of deficits that can occur following prenatal alcohol exposure (PAE), including impaired neurocognitive functioning, emotion and behaviour regulation, and in a smaller proportion of cases, sentinel facial features and growth restriction [1–3]. High rates of early childhood adversity and adverse outcomes are also frequently reported in this population, and among these, overrepresentation in the criminal justice system is perhaps among the most costly and impactful to individuals and society [4–9]. Coupled with 90% estimated rates of mental health comorbidity, and the addition of FASD to the *Diagnostic and Statistical Manual of Mental Disorders* (DSM-5) as both an exemplar of “Other Specified Neurodevelopmental Disorder,” and a condition for further study, clinicians are increasingly likely to encounter individuals with FASD, particularly in the criminal justice context [6, 10].

Epidemiological estimates drawn from limited empirical data suggest that many as 60% of adolescents and adults with FASD assessed through clinical settings have contact with the criminal justice system, a rate 30 times higher than the general population [5, 11, 12]. Canadian prevalence data from two unpublished research reports focusing on justice-involved adults using active case ascertainment methods place rates of FASD between 10 and 17% [13, 14], compared to between 2 and 5% in the general North American population [12, 15–18, 19]. Two published studies have estimated FASD prevalence in justice-involved youth using similar approaches in both Canada (23%) and Australia (36%), underscoring concerns regarding overrepresentation [20, 21]. These estimates are all considered conservative, given the challenges inherent in confirming PAE in justice-involved populations in both clinical and research contexts. These challenges include lack of evidence regarding the ultimate phenotypic presentation of adults with FASD, including those in the criminal justice system, challenges inherent in identifying cases in the context of varied neurocognitive presentations that often go undetected, difficulties confirming PAE with increasing age, and lack of FASD-related knowledge and training in health professionals working in forensic and correctional settings [22–24].

Limited evidence about FASD prevalence in the criminal justice context hampers our ability to inform the development and effective implementation of supportive health practices, including screening, assessment, diagnosis, and intervention. Furthermore, failure to identify and attend to the specialized neurocognitive, criminogenic, and health needs of this population likely contributes to increased recidivism and victimization risk. In turn, this may further entrench criminal justice system involvement, which is linked with poor health, social, and economic outcomes [25–27, 28]. There is also increasing recognition that the neurocognitive deficits, and associated behavioural and mental health comorbidities, are relevant in a range of forensic and adjudicative contexts, including arrest warning comprehension and the validity of statements provided during police interrogation, fitness to plead and stand trial, criminal responsibility, long-term and dangerous offender designations, violence risk assessment, sentencing considerations, and transition planning for discharge back to community [24, 25, 29, 28]. Thus, the current study sought to estimate the prevalence of FASD in justice-involved adults in a northern Canadian correctional jurisdiction, providing important information for clinicians, administrators, and policy makers, in understanding the relevance of FASD across multiple health and legal contexts.

## Method

### Study design

We used an active case-ascertainment approach wherein we recruited a representative sample of justice-involved adults on current legal supervision orders (e.g., bail, probation, remand, sentenced custody, community wellness court). Data collection was conducted over an 18-month period between 2013 and 2015. The total annual population of justice-involved adults between the ages of 18 and 40 was approximately 450 individuals in the study jurisdiction.

### Ethics

The study was approved by the research ethics board at the University of British Columbia Child and Women’s Research Ethics Board and adhered to governing ethical guidelines. A study oversight committee comprising local and national experts, and community, First Nations, and government stakeholders oversaw all study procedures. An enhanced approach to informed consent was used to ensure participants and potential collateral informants had sufficient comprehension prior to providing written informed consent and consent to access records. Participants were provided monetary incentives for study completion considered commensurate with the time required to complete the study. Participants were offered individual feedback sessions and lay language reports characterizing their diagnostic outcome, personal strengths and limitations, and recommendations. A research team member remained in place for six months following study completion to ensure participants could contact the team for follow-up support.

#### Recruitment

Several recruitment approaches were combined to achieve a representative sample. Probation officers and case managers approached all eligible clients about the study during the study enrolment period, and with permission from interested potential participants, made direct referrals to the research team. The research team facilitated study information sessions in community and custody settings, and the study was widely advertised, facilitating self-referral.

#### Eligibility

All individuals ages 18 to 40 on an active justice supervision order for at least three weeks following study enrolment were eligible to participate.^1^ The upper age restriction was consistent with previous similar studies, and put in place in order to maximize likelihood of receiving information about PAE, while minimizing increasingly complex challenges related to differential diagnosis in aging offender populations [13, 14, 22]. Individuals who were considered medically or psychiatrically unstable were also excluded.

#### Representativeness

**Fig. 1:**
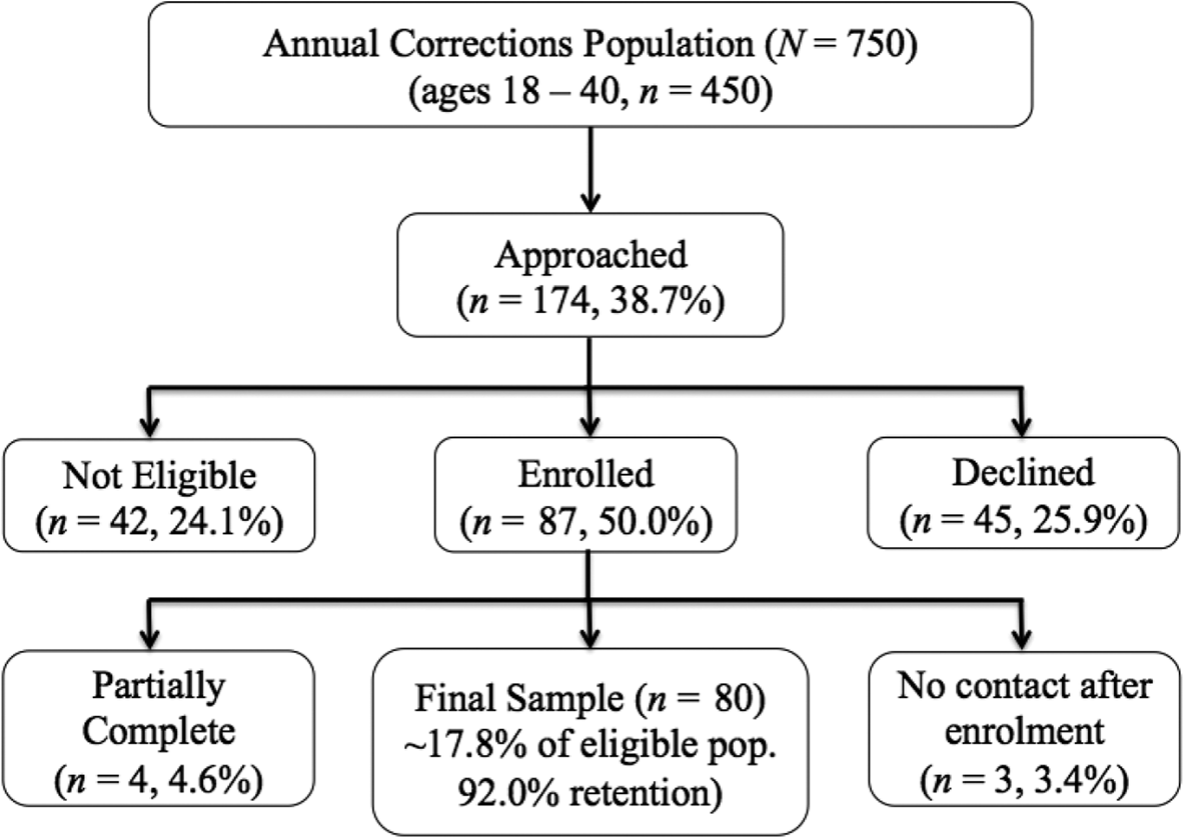
Recruitment, retention, and sample representativeness

The research team approached 174 prospective participants. In total, 42 individuals (24.1%) were ineligible (mainly due to age), 45 declined (25.9%) with frequently cited reasons including, time commitment, low compensation, and lack of interest, and 87 (50.0%) consented to participate. Seven cases were excluded from final analyses owing to partial assessments (8% attrition). The final sample reflected approximately 17.8% of the annual eligible correctional population (e.g., adults ages 18–40, Fig. 1).

#### Participants

Our final sample included 80 justice-involved adults. (Table 1). Consistent with local correctional census data, participants were predominantly male. There was also a notable overrepresentation of participants reporting Indigenous^2^ heritage that was consistent with their respective overrepresentation among incarcerated adults in the study jurisdiction. Most participants were awaiting adjudication on at least one charge and were recruited while in custody, though many shifted between custody and community living during participation. Most had achieved an education level below high school completion (e.g., < grade 12), and nearly half reported a history of foster care.

**Table 1:** Sample Characteristics (N = 80)

	n (%)		n (%)
Age (*M,* SD)	29.38 (5.34)	Completed Gr. 12/GED	19 (23.8)
Gender (% male)	68 (85.0)	Adjudication Status	
Ethnicity		Pre-adjudication	50 (62.5)
Indigenous^a^	62 (77.5)	Sentenced	30 (37.5)
Caucasian	18 (22.5)	Custody Status	
Marital Status		Custody	70 (87.5)
% Single	53 (66.3)	Community	10 (12.5)
History of Foster Care	34 (42.5)		

^a^First Nation, Métis, Inuit

### Assessment and classification approach

Diagnostic procedures adhered to the 2005 Canadian Diagnostic Guidelines for FASD.^3^ Participants completed a multidisciplinary diagnostic assessment involving a physician, psychologist, and clinical coordinator. Assessment procedures involved a semi-structured interview canvasing personal, social, and medical history, analysis of three digital facial photographs for sentinel facial features [30], medical assessment, and a comprehensive psychological evaluation. The clinical research team received training, supervision, and consultation from clinicians with FASD expertise in medicine and neuropsychology. Nine neurodevelopmental domains were assessed using a range of measures (Table 3).

PAE was assessed based on file review, maternal, and collateral reports. More than 300 unique records from child welfare, education, justice, and health practitioner sources, were requested (with written consent from participants) yielding a large volume of historical information about participants and in some cases birth mothers. We also conducted interviews with 34 birth mothers and 11 collateral informants and used maternal and collateral report versions of the Brief Screening Checklist (see measures) to canvass information about potential alcohol exposed pregnancies and additional exposure to drugs. All available information was reviewed by the diagnostic team who came to a clinical consensus and ranked PAE using the four-digit diagnostic code [31] (e.g., confirmed absence of exposure from birth; unknown exposure (neither confirmed absent nor confirmed present); confirmed exposure, but level is below high, or unknown; and, confirmed exposure to high levels of alcohol).

FASD features (e.g., growth, face, brain, and PAE) were ranked and/or considered ‘impaired’ according to recommended cut-off scores (Table 2). Clinical information was reviewed at case conferences and used to make diagnoses, including fetal alcohol syndrome (FAS), partial fetal alcohol syndrome (pFAS), and alcohol related neurodevelopmental disorder (ARND). A diagnostic ‘deferral’ was made for cases where diagnostic criteria were met in the absence of reliable information about PAE.

**Table 2:** Diagnostic criteria for FASD using the 2005 Canadian Diagnostic Guidelines [3]

	Growth	Face	CNS	PAE
FAS	Evidence of prenatal growth impairment, as in at least 1 of: - Birth weight/length ≤ 10th percentile for GA - Height/weight ≤ 10th percentile for age - Disproportionately low weight-height ratio (= 10th percentile)	Simultaneous presentation of all 3 sentinel facial features - Short palpebral fissure length (≤ 2 SD below mean) - Smooth or flattened philtrum (rank 4 or 5)^a^ - Thin upper lip (rank 4 or 5)^a^	Evidence of impairment in ≥3 CNS domains:	Confirmed or unconfirmed PAE
pFAS	–	Simultaneous presentation of 2 sentinel facial features (as above)	Impairment in ≥3 CNS domains	Confirmed PAE
ARND	–	–	Impairment in ≥3 CNS domains	Confirmed PAE

*FAS* Fetal Alcohol Syndrome, *pFAS* partial Fetal Alcohol Syndrome, *ARND* Alcohol Related Neurodevelopmental Disorder, *GA* Gestational Age, *PAE* Prenatal alcohol exposure, *CNS* Central Nervous System. ^a^Philtrum and upper lip were ranked following the 4-digit diagnostic code and lip/philtrum guide [33]

### Measures

#### Medical intake interview

We used a modified semi-structured medical and social history interview developed for FASD research in the correctional context that canvassed physical and mental health, and pertinent family and social history [13, 14].

#### FAS facial photographic analysis software

[30] Three digital facial photographs (frontal, 3/4, and lateral views) were taken and analyzed using the Facial Photographic Analysis Software (Canadian norms [32]) to classify the magnitude of expression of key diagnostic facial features (short palpebral fissure lengths, thin upper lip) following the 4-digit diagnostic code [33]. Philtrum depth was manually assessed using the lip/philtrum guide [33].

#### Neurodevelopmental and cognitive assessments

Participants completed a comprehensive psychological test battery assessing functioning across neurodevelopmental domains (Table 3). Measures were selected based on sound psychometric properties, consensus guidelines on use of psychometric tools for evaluating individuals with FASD, the 2005 Canadian Diagnostic Guidelines [3], and the availability of appropriate normative data for the study jurisdiction.

**Table 3:** Measures used to assess neurobehavioural domains in the FASD assessment

Domain	Measures
Hard and Soft Neurological Signs	Medical intake interview Medical assessment Adolescent/Adult Sensory Profile [44]
Brain Structure	Medical intake interview Medical assessment Head circumference
Cognition (IQ)	Wechsler Adult Intelligence Scale-IV (WAIS-IV) [45] Subtests: Similarities, Vocabulary, Information, Block Design, Matrix Reasoning, Visual Puzzles, Digit Span, Arithmetic, Letter-Number Sequencing, Symbol Search, Coding
Attention	Connors’ Continuous Performance Test – II (CPT-II) [46]
	Wechsler Adult Intelligence Scale-IV (WAIS-IV) [45] Subtests: Digit Span
Academic Achievement	Wide Range Achievement Test – Fourth Edition (WRAT-4) [47] Subtests: Word Reading, Sentence Comprehension, Spelling, Math Computation
Memory	Wechsler Memory Scale – 4th Edition (WMS-IV) [48] Subtests: Logical Memory I & II, Design Memory I & II
	California Verbal Learning Test – 2nd Edition (CVLT-II) [49]
	Rey Osterreith Complex Figure Test (RCFT) [50]
Language/ Communication	Wechsler Adult Intelligence Scale-IV (WAIS-IV) [45] Subtests: Vocabulary, Similarities, Information Medical Intake Interview
Executive Functioning	Delis Kaplan Executive Function System (DKEFS) [49] Subtests: Sorting, Color-Word Interference, Verbal Fluency, Design Fluency, Trails
Adaptive Functioning	Adaptive Behavior Assessment System – Second Edition (ABAS-II) [51] Subtests: Conceptual, Social, and Practical domains

### Statistical analyses

Descriptive statistics are presented to characterize the sample for continuous (e.g., means, standard deviations) and categorical data (counts, percentages). Group comparisons were made using Analysis of Variance (ANOVA) and Chi-squared tests. Effect sizes (phi coefficient, partial eta squared) and 95% confidence intervals are reported. Statistical analyses were conducted using IBM SPSS version 24.0 for Mac. We conducted a priori power calculations using parameters for precision (.8) and power (.95), in a finite population (450), and an estimated point prevalence of .20 based available criminal justice prevalence estimates, local clinic data, and expert input using the approach outlined in Daniel (1999) and Naing et al., (2006) [31, 34]. Results suggested that our final sample would provide sufficient detection power for a reliable prevalence estimate while balancing practical limitations inherent in conducting diagnostic research in a setting marked by limited clinical resources [31, 34].

## Results

### FASD diagnosis

In total, 14 individuals received an FASD diagnosis (pFAS or ARND) yielding an estimated prevalence of 17.5% (95% CI [9.2, 25.8]) (Table 4). Of these individuals, only two had been previously diagnosed (14.3%). Diagnostic decisions could not be made with reliability in 11 cases (13.8%, considered ‘deferred’), indicating that our prevalence estimate could have been as high as 31.2% with sufficient information about PAE.

**Table 4:** Diagnostic outcomes compared to Canadian federally incarcerated adult offenders

	Current Sample (*N* = 80) *n* (%)	Justice-involved Adult Men [14] (*N* = 91) *n* (%)	Justice-involved Adult Women^a^ [13] (*N* = 23) *n* (%)
Diagnostic Outcomes			
FASD	14 (17.5)	9 (10.0)	4 (17.4)
FAS	0 (0.0)	0 (0.0) 1 (1.2)	0 (0.0)
pFAS	2 (2.5)		1 (4.3)
ARND	12 (15.0)	8 (8.8)	3 (13.0)
Deferred	11 (13.8)	–	–
Not Diagnosed	55 (68.8)	–	–

^a^ Due to limitations in the availability of reliable information stemming from neuropsychological assessment and confirmation of PAE, diagnostic classifications were reported as “probable” rather than “confirmed.” *FASD* Fetal Alcohol Spectrum Disorder, *FAS* Fetal Alcohol Syndrome, *pFAS* Partial Fetal Alcohol Syndrome, *ARND* Alcohol Related Neurodevelopmental Disorder

#### Prenatal alcohol exposure

PAE was confirmed for 20 participants (25.0%), with all but one case classified at ‘some risk’.^4^ PAE was considered reasonably ruled out for a further 20 participants based on information collected from multiple sources (25.0%), and considered ‘unknown’ for the remaining 40 participants (50.0%), based on limited to no available relevant information across multiple sources. Six participants with confirmed PAE did not receive a diagnosis owing to insufficient detected impairment across neurodevelopmental domains.

#### Growth

Current growth was considered within normal limits for most participants (*n* = 77, 96.2%), and none of the cases with ‘mild’ to moderate’ growth deficiency received a diagnosis.

#### Facial features

Facial features in the ‘mild’ and ‘moderate’ ranges (rank 2 or 3) were identified for 14 participants (17.5%), though none presented with all three sentinel features simultaneously. Of these, two participants were diagnosed with pFAS (14.3%), and five were deferred (35.7%). Smooth philtrum was present (e.g., rank 4 or 5) in six cases (7.5%, two diagnosed, one deferred). Ten participants (12.5%) had evidence of thin upper lip (e.g., rank 4 or 5, one diagnosed, two deferred). Six participants (7.5%) had mean palpebral fissure lengths shorter than two standard deviations below the mean (none diagnosed, one deferred). Overall, there were no significant differences on physical indicators of PAE between diagnostic groups.

#### Neurodevelopmental dysfunction

Five participants (6.3%) were considered free of significant neurodevelopmental impairment across domains (see Table 3). More than half (*n* = 44, 55.0%) were assessed as having ‘moderate’ dysfunction (e.g., significant impairment in at least two domains), while the remaining third (*n* = 31, 38.7%) had ‘severe dysfunction’ (e.g., significant impairment in at least three domains).^5^ Impairment within each of the six neurodevelopmental domains was also rated on a three-point scale, including ‘no impairment,’ ‘mild to moderate impairment,’ and ‘significant impairment.^6^ Diagnosed and deferred participants had significant impairment in a greater number of neurodevelopmental domains compared to those who were not diagnosed, *F* = 49.93, *p* < .001, η^2^_p_ = .57 (Table 5). Notably, more diagnosed participants had significant impairment in the executive functioning domain (*n* = 10, 71.4%) relative to those who were deferred (*n* = 3, 27.3%) and not diagnosed (*n* = 3, 5.5%). One third of participants (*n* = 27, 33.8%) had IQ scores below common clinical cut points (e.g., < 70) used to establish a diagnosis of intellectual disability.

**Table 5:** Physical, cognitive, PAE, and health features by diagnostic outcome

	FASD				Deferred				Not Diagnosed			
Four-Digit Code [*n,* (%)]	1	2	3	4	1	2	3	4	1	2	3	4
Growth	14 (100)	0 (0)	0 (0)	0 (0)	10 (91)	1 (9)	0 (0)	0 (0)	53 (69)	1 (2)	1 (2)	0 (0)
Face	12 (86)	2 (14)	0 (0)	0 (0)	6 (54)	5 (46)	0 (0)	0 (0)	48 (87)	6 (11)	1 (2)	0 (0)
CNS	0 (0)	0 (0)	14 (100)	0 (0)	0 (0)	1 (9)	10 (91)	0 (0)	5 (9)	43 (78)	7 (13)	0 (0)
PAE	0 (0)	0 (0)	12 (86)	2 (14)	1 (9)	10 (91)	0 (0)	0 (0)	19 (35)	30 (55)	5 (9)	1 (2)
Medical findings												
Height (cm) *M* (SD)	176.5 (4.7)				175.4 (6.9)				174.7 (8.3)			
Weight (kg) *M* (SD)	87.5 (15.4)				93.8 (20.1)				84.6 (18.3)			
BMI *n* (%) obese	4 (29)				5.0 (46)				13.0 (24)			
Occipital Circ. (cm) *M* (SD)	56.9 (1.9)				57.1 (1.6)				57.0 (2.0)			
Palpebral fissure length (mm) *M* (SD) [*M z*-score (SD)]	29.0 (1.8) [.4 (1.2)]				28.3 (2.2) [−.1 (1.5)]				28.7 (2.2) [.2 (1.5)]			
Intercanthal distance (mm) *M* (SD) [*z*-score]	33.6 (3.3) [1.1 (1.1)]				35.0 (3.1) [1.5 (1.3)]				33.3 (3.4) [.8 (1.4)]			
Smooth philtrum % Rank 4/5 *n* (%)	2 (14)				1 (9)				2 (4)			
Thin vermillion border *M* (SD) [*n* (%) Rank 4/5]	53.7 (16.7) [2 (14)]				67.0 (23.0) [4 (36)]				59.6 (14.2) [5 (9)]			
Ear anomalies *n* (%)	0 (0)				0 (0)				5 (9)			
Palate anomalies *n* (%)	3 (21)				4 (36)				5 (9)			
Poor dentition *n* (%)	5 (38)				5 (46)				14 (26)			
Spine anomaly *n* (%)	2 (15)				1 (9)				3 (6)			
Current medications *n* (%)	6 (43)				5 (46)				28 (51)			
Past suicide attempt *n* (%)	6 (43)				5 (46)				22 (40)			
Previous Head Injury *n* (%)	11 (79)				8 (73)				30 (55)			
Cognitive findings												
Cognition *n* (%) sign. Imp.	11 (79)				9 (82)				14 (26)			
IQ *M* (*SD*)^a^	65.4 (5.4)				68.5 (4.7)				83.1 (12.3)			
IQ < 70 *n* (%)^a^	10 (77)				7 (64)				10 (19)			
Attention *n* (%) sign. Imp.	6 (43)				5 (50)				12 (13)			
Academics	6 (43)				4 (36)				1 (2)			
Memory	6 (43)				4 (36)				6 (11)			
Executive function	10 (71)				3 (27)				3 (6)			
Adaptive function	14 (100)				11 (100)				50 (91)			
# domains sign. Imp. /6 *M* (*SD*)	3.8 (.2)				3.3 (.3)				1.6 (.1)			

*N* = 80. *CNS* Central Nervous System, *PAE* Prenatal Alcohol Exposure. ^a^*N* = 77 as raw IQ scores were not available for three participants who had completed psychological testing elsewhere in the last year

#### Additional findings

Although not systematically assessed, many participants reported sleep problems, and a high number reported a history of head injury resulting in loss of consciousness (*n* = 49, 61.3%). Body mass index (BMI) scores in the ‘obese’ range (e.g., BMI ≥ 30) were observed in approximately one-quarter of participants (*n* = 22, 27.5%). Approximately half the sample reported taking medication (*n* = 39, 48.8%), including antidepressants (*n* = 14, 17.5%, SSRIs, tricyclics, and tetracyclics), melatonin (*n* = 10, 12.5%), stimulant ADHD medications (*n* = 5, 6.3%, e.g., methylphenidate, dextroamphetamine), atypical antipsychotics (*n* = 5, 6.3%, e.g., Risperidone, Quetiapine), and anti-epileptic medications (*n* = 5, 6.3%, e.g., gabapentin, pregabalin). Rates of palate abnormalities were high (*n* = 12, 15.0%) along with poor overall dentition (*n* = 30, 37.5%), highlighting the range of health difficulties present in this population. Participants also reported high rates of past suicide attempts across diagnostic groups (*n* = 33, 41.2%).

In addition, most participants reported experiencing at least one episode of lifetime abuse or neglect (*n* = 66, 83% for any experience of physical, sexual, emotional abuse, or neglect), with most participants also reporting multiple abuse/neglect experiences, and high rates of both physical (*n* = 48, 60%), and sexual abuse (*n* = 34, 43%) across the sample. Although the conservative nature of our sample precludes detailed analysis, it is noteworthy that all women in the current study reported experiencing prior abuse or neglect, accounting for a large proportion of the victimization experiences characterized within the current sample.

## Discussion

This study represents one of few to systematically estimate rates of FASD in justice-involved adults in Canada. In total, 17.5% of 80 adults ages 18 to 40 in a northern Canadian correctional jurisdiction were diagnosed with FASD using an active case ascertainment approach and following the Canadian Guidelines for FASD.^3^ [3] Results were consistent with other Canadian studies using prospective clinical assessments, including two reports that estimated FASD prevalence in federally sentenced adult men (9.9% of 91 participants) and women (17.0% of 23), and one study of youth in an inpatient forensic psychiatric program (23.3% of 287 remanded youth) [13, 14, 20]. Together, these findings contribute to a growing evidence base highlighting high FASD rates in a range of Canadian geographic locations and correctional contexts (e.g., 10 to 17% in adults). Taking even the midpoint of this range (e.g., 13.7%) this number is far greater than prevalence estimates for the general population (e.g., 2 to 5%), and represents a major public health concern [12, 15, 16].

There are also several reasons to suggest that the current finding reflects an underestimate of FASD prevalence in the study jurisdiction. First, in the event that PAE had been confirmed present above risk thresholds for our 11 deferred cases, the estimate could have been as high as 31.2%. Second, we did not consider the affect regulation domain, now included in the 2015 Canadian FASD Diagnostic Guidelines [2]. Given the high rate of mental health related difficulties in this population and in corrections broadly, this could have resulted in a greater number of diagnosed cases (e.g., in six cases, PAE was confirmed but testing did not detect sufficient neurobehavioural impairment for diagnosis). In addition, despite our broad and thorough medical and neuropsychological evaluation, additional clinical investigation may have yielded ‘hits’ in additional domains, such as with the presence of a more detailed speech language pathology assessment.

In the current study, most individuals diagnosed with FASD had not been previously identified (86%), consistent with similarly high rates of missed diagnosis reported in Australian and Canadian youth justice and forensic samples [20, 21]. This, coupled with the finding that 93.6% of our sample had neurocognitive impairment in at least one domain, and 38.8% demonstrating significant impairment in three or more domains, suggests that there are a high number justice-involved adults with unidentified complex cognitive needs. Indeed, one-third of our sample was assessed as having an IQ score < 70, the cut-off frequently used for diagnosis of Intellectual Disorder [10]. In addition to the range of reported health needs and exceptionally high rates of early adversity and trauma, our findings highlight the inherent vulnerability of this population and begs important questions about whether their ultimate involvement in the criminal justice system could have been averted if earlier and appropriate assessment and interventions had been provided in other systems (e.g., health, education., etc.). In addition, results underscore the need for proactive and systematic consideration of FASD in the context of broad and comprehensive mental and physical health assessments early on in adjudicative and correctional contexts. Given that half our sample had pending charges, the importance of access to timely comprehensive evaluations that include FASD, may allow the courts to provide meaningful sentences with appropriate conditions to enhance positive rehabilitative outcomes.

Results from this study complement those from other justice-involved samples marked by high rates of ARND, few cases of pFAS and FAS, and therefore limited evidence of overt physical indicators linked with PAE. This result departs from many studies highlighting the salience of these indicators in clinically-referred samples of children and youth, and suggests the need for continued research in this context. (e.g. [35, 19],) Furthermore, this finding may suggest that screening and/or assessment strategies that focus primarily on facial features, or growth, may result in continued under-identification of individuals at risk of having FASD and/or similarly complex neurocognitive presentations, particularly in the adult criminal justice context [13, 14]. Our findings suggest the importance of broad consideration of neurocognitive abilities and needs in this respect. It is also possible that there are important and clinically meaningful differences between individuals presenting to community-based clinics, and adults assessed in a criminal justice context. For instance, Streissguth and colleagues found that individuals diagnosed with fetal alcohol effects (FAE) were at greater risk of getting into trouble with the law compared to those diagnosed with FAS. They questioned whether individuals who do not present with physical indicators associated with FASD may be at greater risk of going undetected, and as a result, may not be provided necessary services and supports in the context of substantial neurobehavioural impairment [5]. Clarification of these potentially meaningful differences may help to better understand and inform risk and resilience trajectories in this population.

### Strengths and limitations

This study had several strengths, including use of an active case ascertainment approach, following an interdisciplinary clinical research model, using a comprehensive and uniform assessment battery, developing FASD clinical and research capacity in the study jurisdiction, and providing participants with individualized feedback and supports. However, several limitations also warrant review. First, our sample likely best generalizes to similar northern Canadian correctional populations and the reliability of our estimate may be limited by a conservative sample size and voluntary participation. As anticipated, confirmation of PAE proved a challenge in this non-clinically referred adult sample, likely mirroring challenges in ‘real world’ clinical and justice contexts for adults. Taken together, there is a clear need for further research and replication using larger samples and across locations (e.g., larger metropolitan areas). This will be particularly important in the context of the new Canadian FASD diagnostic guidelines [2].

In keeping with previously published correctional prevalence studies, our sample included a high number of Indigenous Canadians. It is critical to frame these findings in the context of factors that contribute to high rates of FASD (e.g., social determinants of health) in vulnerable populations, and to reinforce the point that FASD occurs in all populations where alcohol is used, irrespective of the ethnicity of community members [36, 37]. In this study jurisdiction, like many places in Canada and worldwide, Indigenous communities continue to recover from colonialist policies, leading to intergenerational impacts that include a loss of cultural identity, weakening of community and family integrity, and disproportionate rates of trauma, poverty, addiction, and poor health. Understanding the current results through this lens should inform a range of policies and solutions geared toward supporting the health and wellbeing of vulnerable individuals and communities in their recovery, both in community settings, and also specifically in the context of the criminal justice system [38]. Furthermore, the overrepresentation of Indigenous Canadians in our sample underscores the need to develop and implement collaborative health evaluations, including neuropsychological and medical assessment, that take into consideration diverse conceptualizations of health and wellbeing, and culturally safe approaches to strength-based assessment and intervention [39].

## Conclusions

This study adds to the limited but growing body of evidence demonstrating overrepresentation of individuals with FASD in criminal justice settings. However, several factors currently limit system capacity to provide an effective response to the problem. These include lack of training and awareness about FASD among clinicians working in criminal justice contexts, limited diagnostic capacity, lack of evidence-based screening tools, and the relative “invisibility” of FASD [40, 41]. Consistent with previous studies, our findings suggest that most individuals with FASD in the justice system went undetected. Unaddressed, the impact of PAE coupled with early life adversity, complex cognitive deficits, and other physical and mental health comorbidities, may lead to inadequate personal medical care, and difficulty benefitting from traditional correctional intervention approaches. There is a clear lack of community-based services for individuals with FASD in Canada, and combined, these factors may lead to increased recidivism and entrenchment in the criminal justice system, as well as high risk of victimization in this population [25, 42, 43].

Critical future research directions should focus on understanding the needs, antecedents, and trajectories through the criminal justice system for individuals with FASD to inform evidence-based clinical and correctional intervention approaches. Our findings also underscore the importance of adopting a ‘prevention lens’ to ensure that children, families, and communities have necessary supports required to avoid problematic criminal justice system involvement. Turning our focus toward the development of integrated and intersectoral policy solutions will prove critical in reducing the overrepresentation of individuals with FASD in the criminal justice system.

## Abbreviations

ADHD: Attention Deficit Hyperactivity Disorder
ANOVA: Analysis of Variance
ARND: Alcohol related neurodevelopmental disorder
BMI: Body mass index
BSC: Brief Screening Checklist
DSM-5: Diagnostic and Statistical Manual for Mental Disorders
FAS: Fetal alcohol syndrome
FASD: Fetal alcohol spectrum disorder
PAE: Prenatal alcohol exposure
pFAS: Partial fetal alcohol syndrome

## Footnotes

1 Individuals serving Federal sentences or under the supervision of a Review Board were not eligible

2 Individuals identifying their background as First Nation, Métis, or Inuit.

3 A revised set of Canadian Diagnostic Guidelines for FASD were released subsequent to data collection, resulting in changes to diagnostic terminology and criteria [2].

4 PAE was ranked using the 4-digit diagnostic code [33]. Rank 4 (‘high risk’) is rendered in cases of confirmed high risk PAE, Rank 3 (‘some risk’) is reserved for cases where PAE is confirmed but level is considered less than rank 4 or unknown, Rank 2 (‘unknown risk’) is used when PAE is unknown, and rank 1 (‘no risk’) is given when PAE is confirmed to be completely absent.

5 Clinicians ranked the presence of neurodevelopmental deficits across all domains using the 4-digit diagnostic code (Rank 1 = No Dysfunction; Rank 2 = Moderate Dysfunction; Rank 3 = Severe Dysfunction) (Table 5). [33]

6 Hard and soft neurological signs, brain structure, and communication were not rated in this manner owing to more circumscribed measurement in these areas.
